# Correction: Smurf1 inhibits integrin activation by controlling Kindlin-2 ubiquitination and degradation

**DOI:** 10.1083/jcb.20160907302062026c

**Published:** 2026-02-13

**Authors:** Xiaofan Wei, Xiang Wang, Jun Zhan, Yuhan Chen, Weigang Fang, Lingqiang Zhang, Hongquan Zhang

Vol. 216, No. 5 | https://doi.org/10.1083/jcb.201609073 | April 13, 2017

The authors discovered that the Actin blots on the left side of Fig. 8 A had been inadvertently duplicated and flipped on the right side. The original and corrected Fig. 8 are shown here. The figure legend remains unchanged.

This error has been corrected but appears in print and in PDFs downloaded on or before February 9, 2026. The authors apologize for any confusion this may have caused.

**Figure:**
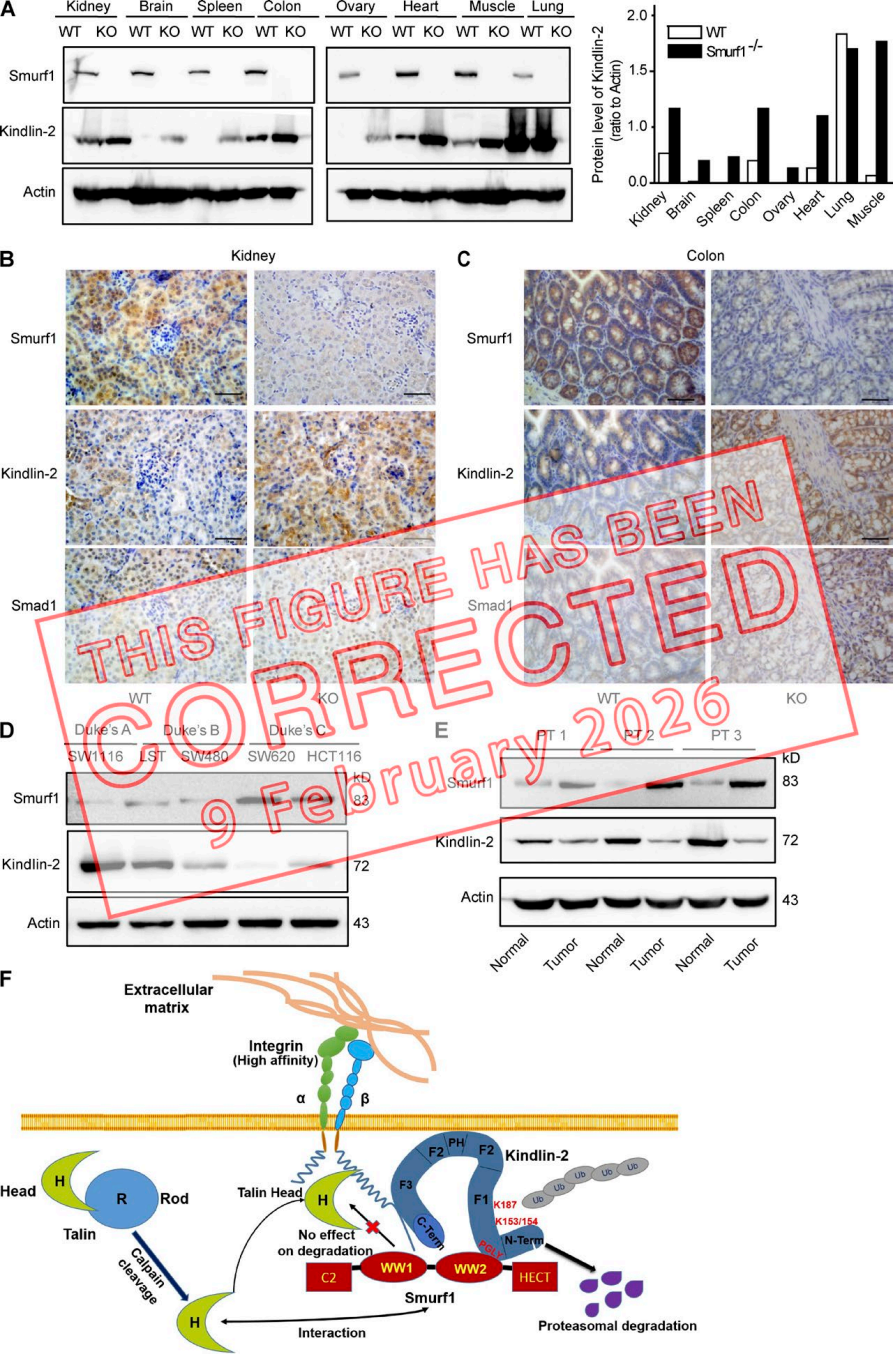


**Figure 8.**
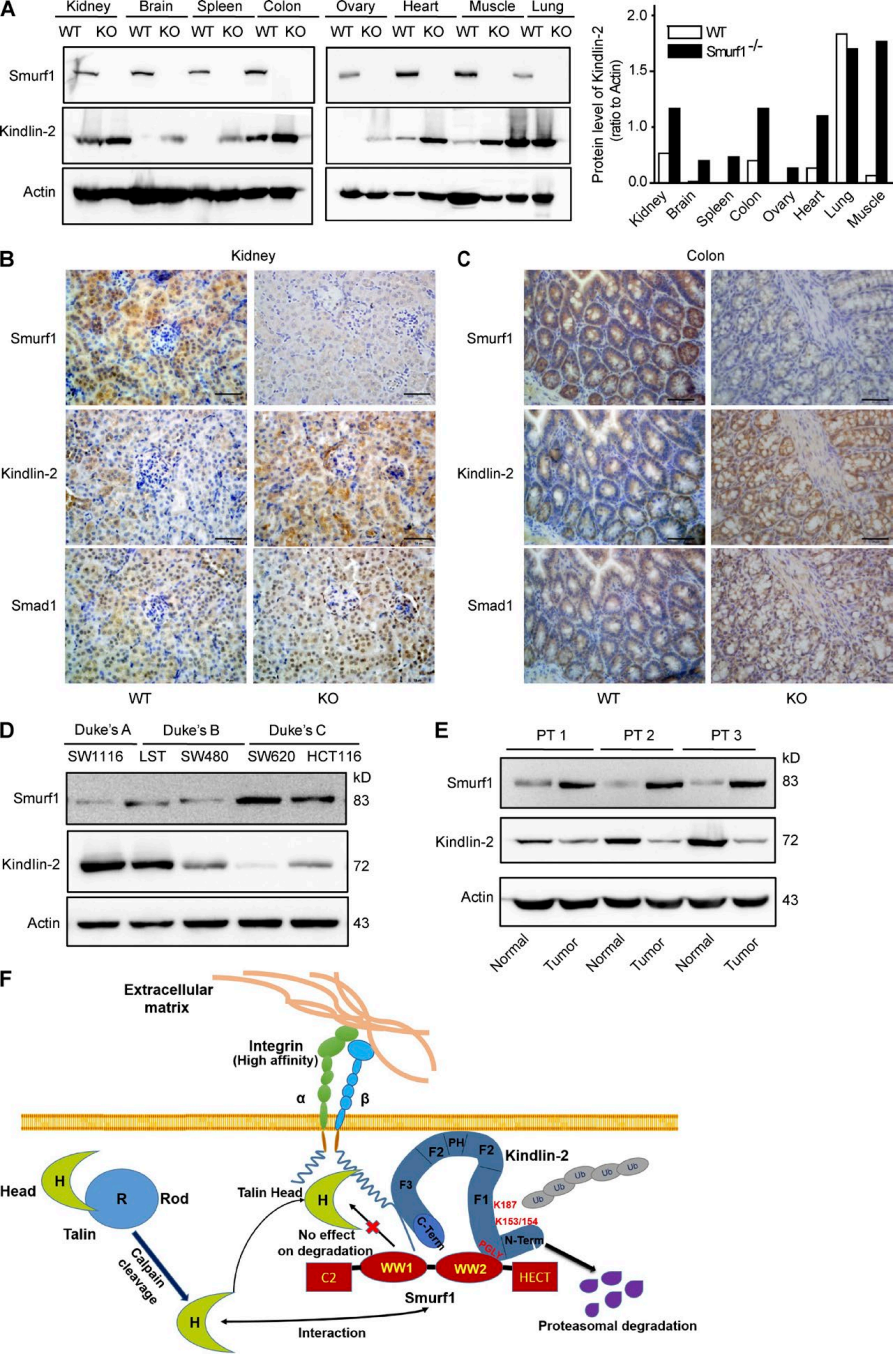
**The expression of Smurf1 is negatively related with Kindlin-2 expression in vivo.** (A) Endogenous Smurf1 and Kindlin-2 protein expression was detected in indicated organs tissues of WT or Smurf1^−/−^ mice by Western blot. (B and C) Representative immunohistochemical micrographs showing the expression of Smurf1, Kindlin-2, and Smad1 in the kidney and colon tissues of WT, Smurf1, or Smurf1^−/−^ mice. Bars, 50 µm. (D) Smurf1 and Kindlin-2 protein expression in diverse colon cancer cell lines examined by immunoblotting. (E) Smurf1 and Kindlin-2 protein expression in three human colon cancer tissues was determined by Western blot. (F) A hypothetical model for Smurf1 modulation of integrin activation. Both Talin and Kindlin-2 stimulate integrin activation via Talin-H and Kindlin-2 FERM domain binding to integrin β cytoplasmic tail. Smurf1 directly interacts with Kindlin-2 through the Smurf1-WW2 domain and the PY-motif in Kindlin-2. Smurf1 mediates Kindlin-2 polyubiquitination, leading to the proteasomal degradation of Kindlin-2, thereby inhibiting integrin activation. Although Smurf1 interacts with Talin-head, Smurf1 does not mediate the degradation of Talin-H or the full-length Talin. Therefore, Smurf1 does not influence integrin activation mediated by Talin alone. Collectively, Smurf1 controls proper integrin activation by interacting with and limiting the amount of Kindlin-2, a helper in Talin-mediated integrin activation.

